# Deciphering the hydrogen-bonding scheme in the crystal structure of tri­phenyl­methanol: a tribute to George Ferguson and co-workers

**DOI:** 10.1107/S2053229619010714

**Published:** 2019-08-14

**Authors:** Tomasa Rodríguez Tzompantzi, Aldo Guillermo Amaro Hernández, Rosa Luisa Meza-León, Sylvain Bernès

**Affiliations:** a Facultad de Ciencias Químicas, Benemérita Universidad Autónoma de Puebla, Av. San Claudio y 18 Sur, 72570 Puebla, Pue., Mexico; b Instituto de Física, Benemérita Universidad Autónoma de Puebla, Av. San Claudio y 18 Sur, 72570 Puebla, Pue., Mexico

**Keywords:** alcohol, hydrogen bond, disorder, topological chirality, crystal structure, triphenylmethanol

## Abstract

The crystal structure of tri­phenyl­methanol, C_19_H_16_O, has been redetermined using data collected at 295 and 153 K, and is compared to the model published by Ferguson *et al.* over 25 years ago [Ferguson *et al.* (1992 ▸). *Acta Cryst*. C**48**, 1272–1275] and that published by Serrano-González *et al.*, using neutron and X-ray diffraction data [Serrano-González *et al.* (1999 ▸). *J. Phys. Chem. B*, **103**, 6215–6223]. As predicted by these authors, the hy­droxy groups are involved in weak inter­molecular hydrogen bonds in the crystal, forming tetra­hedral tetra­­mers based on the two independent mol­ecules in the asymmetric unit, one of which is placed on the threefold symmetry axis of the *R*  space group. However, the reliable determination of the hy­droxy H-atom positions is difficult to achieve, for two reasons. Firstly, a positional disorder affects the full asymmetric unit, which is split over two sets of positions, with occupancy factors of *ca* 0.74 and 0.26. Secondly, all hy­droxy H atoms are further disordered, either by symmetry, or through a positional disorder in the case of parts placed in general positions. We show that the correct description of the hydrogen-bonding scheme is possible only if diffraction data are collected at low temperature. The pro­chiral character of the hydrogen-bonded tetra­meric supra­molecular clusters leads to enanti­omorphic three-dimensional graphs in each tetra­mer. The crystal is thus a racemic mixture of ^sup^ *S* and ^sup^ *R* motifs, consistent with the centro­symmetric nature of the *R*  space group.

## Abstract 2

The disordered crystal structure of tri­phenyl­methanol features tetra­hedral chiral clusters formed through weak hydrogen bonds, leading to the formation of three-dimensional supra­molecular motifs having left or right handedness.

## Introduction   

The hy­droxy group is known as one of the most efficient nodes for the formation of hydrogen bonds, as a consequence of the polarization of the O—H bond, and also because it can behave both as donor and acceptor for building intra- or inter­molecular bonds. In this context, the emblematic donor–acceptor mol­ecule is water, and many compounds have been crystallized as hydrates, in which the lattice water mol­ecules contribute to a significant part of the crystal free energy (Batsanov, 2018 ▸); currently, almost 13% of the structures deposited in the Cambridge Structural Database are hydrates (CSD, Version 5.40, updated February 2019; Groom *et al.*, 2016 ▸). The situation is a bit less favourable in the case of alcohols (*R*O—H), especially for tertiary alcohols having the hy­droxy group surrounded by bulky hydro­carbon groups. For example, three hydrates for *tert*-butanol, (CH_3_)_3_COH, have been successfully characterized [namely the dihydrate and hepta­hydrate (Mootz & Stäben, 1993 ▸), and the deca­hydrate (Dobrzycki, 2018 ▸)], while tri-*tert*-butyl­methanol, [(CH_3_)_3_C]_3_­COH, has probably never been crystallized, although it has been studied in the solid state (Malarski, 1974 ▸). Although this mol­ecule is stable, it is not able to form stabilizing inter­molecular O—H⋯O hydrogen bonds, because of the steric hindrance of the three *tert*-butyl groups surrounding the OH donor group (Majerz & Natkaniec, 2006 ▸).
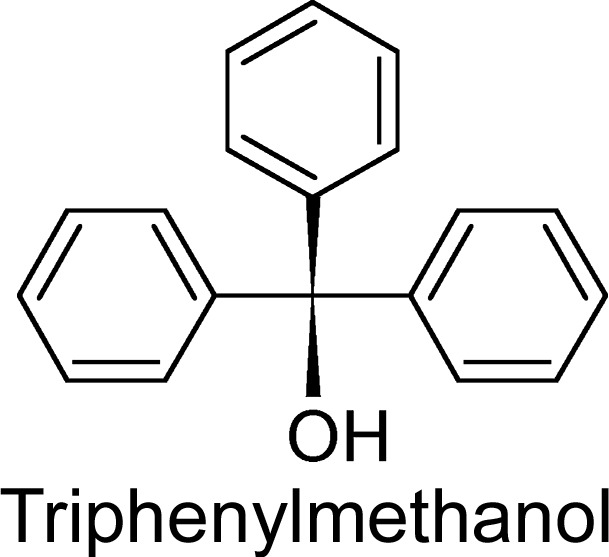


The case of tri­phenyl­methanol, (C_6_H_5_)_3_COH, should be inter­mediate between *tert*-butanol and tri-*tert*-butyl­methanol, since it can be used as a clathrate host for methanol (Weber *et al.*, 1989 ▸), and may be hydrogen bonded to a water mol­ecule (Batisai *et al.*, 2016 ▸), dimethyl sulfoxide, di­methyl­formamide (Eckardt *et al.*, 1999 ▸) or Ph_3_P=O (Steiner, 2000 ▸). Indeed, unsolvated tri­phenyl­methanol can be easily crystallized from benzene or ethanol, affording large well-shaped clear colourless single crystals. However, these crystals are always weakly diffracting samples, as a consequence of a severe structural disorder (*vide infra*). The resulting ratio of observed to measured reflections is then quite low, which, in turn, makes the refinement very difficult. First attempts to refine a reasonable model failed (Weber *et al.*, 1989 ▸), and it was only in 1992 that the crystal structure was published (Ferguson *et al.*, 1992 ▸), based on room-temperature intensities measured on a CAD-4 diffractometer, with Mo *K*α radiation. 2467 unique reflections were used, of which 41% were observed [*I* > 2.5σ(*I*)], and the structure was refined with a structural motif described as a ‘hydrogen-bonded pyramidal tetra­mer which is disordered (71/29) about two inter­penetrating sites’. The refinement was of limited accuracy and converged to *R* = 0.083 and *wR* = 0.068 with rigid idealized phenyl rings for all mol­ecules, and isotropic atoms in the minor-disordered part of the asymmetric unit (253 variable parameters).

Although the structure reported by Ferguson *et al.* was incomplete, since hy­droxy H atoms could not be located, the *savoir-faire* used by this team is quite impressive. They were able to solve and refine this challenging disordered structure, while others probably gave up by arguing that crystals were badly twinned. Above all, they did not attempt to over-inter­pret their data, and were aware that hy­droxy H atoms were very imprecisely determined in their X-ray diffraction experi­ment. However, they indirectly recognized and des­cribed the presence of a weak hydrogen-bonding scheme, reflected in inter­molecular O⋯O contacts.

In 1999, Serrano-González *et al.* (1999 ▸) published a more elaborate article, focussed on the characterization of the hydrogen-bonding arrangement in tri­phenyl­methanol, using neutron (*T* = 100 K) and X-ray diffraction data (*T* = 113 and 293 K), as well as solid-state ^13^C NMR spectroscopy. A reliable structure based on neutron diffraction data was obtained, showing that each independent OH group has the H atom disordered over three sites. This model was then a suitable starting point for the refinement of X-ray structures, both at 113 and 293 K. Unfortunately, the final atomic coordinates were never deposited in the CSD, and there is no CIF available as supporting information. Fractional coordinates are tabulated, however, for X-ray refinements, H atoms are missing. Moreover, even using the favourable neutron scattering length for the protium nucleus, it was not possible to complete the structure. As stated in this article ‘The hy­droxy hydrogens of the minor tetra­mer could not be located from the difference Fourier map, and these hydrogen atoms were inserted in calculated positions based on those determined […] for the major tetra­mer’.

We have now completed these works, using X-ray data collected at room temperature and low temperature with the Ag *K*α radiation, revealing the accurate localization of the hy­droxy H atoms in the disordered structure. A comprehensive insight into the hydrogen-bonding scheme that held together the tetra­meric clusters is now afforded.

## Experimental   

### Synthesis and crystallization   

**Figure 1:**
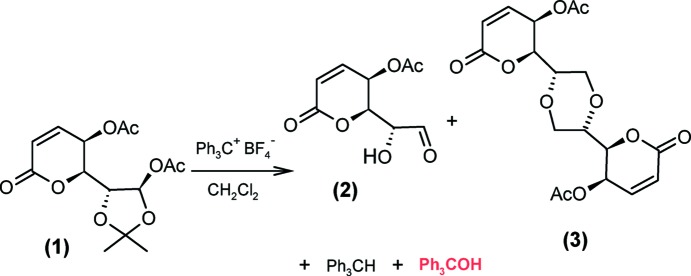
Synthetic step from which tri­phenyl­methanol was crystallized. Note that product (**2**) was not obtained.

We obtained tri­phenyl­methanol as a by-product during the oxidative hydrolysis of the di­acetyl­ated compound (**1**), following a method proposed by Barton *et al.* (1972 ▸) (Fig. 1 ▸). Compound (**1**) (0.100 g, 0.318 mmol) and tri­phenyl­carbenium tetra­fluoro­borate (0.099 g, 0.380 mmol) were mixed in dry CH_2_Cl_2_ (20 ml) and stirred for 15 min at room temperature. A saturated solution of NaHCO_3_ was then added (10 ml), the organic phase dried over Na_2_SO_4_ and the crude product chromatographed (silica gel, hexa­ne–ethyl acetate, 90:10 *v*/*v*). The expected hy­droxy aldehyde (**2**) was not observed, and the dimer (**3**) was isolated instead, mixed with tri­phenyl­methane and tri­phenyl­methanol. It was not possible to separate (**3**) and tri­phenyl­methanol by chromatography, whereby the mixture was purified by crystallization in hexa­ne–ethyl acetate (95:5 *v*/*v*), affording large single crystals of tri­phenyl­methanol [yield 30 mg; m.p. 431–433 K, literature 434–435 K (Zeiss & Tsutsui, 1953 ▸)]. ^1^H NMR (CDCl_3_/TMS, 300 MHz): δ 2.8 (*s*, 1H, OH), 7.30 (*s*, 15H, Ph). ^13^C NMR (CDCl_3_/TMS, 75 MHz): δ 99.8 (C—OH), 127.2, 127.9, 146.8 (Ph).

### Refinements   

**Figure 2:**
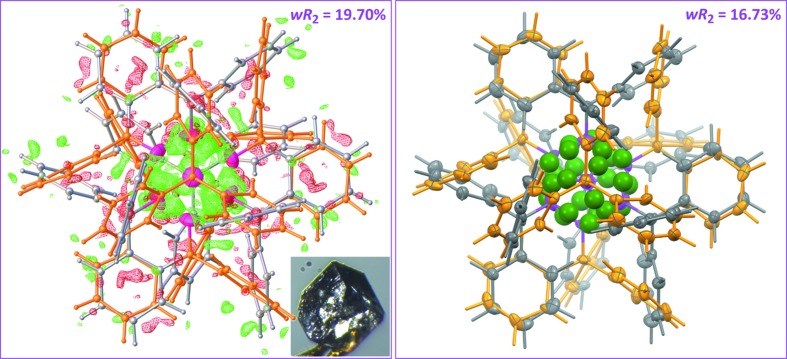
(Left) Difference electron-density map computed using low-temperature data, after refining the full disordered model but omitting hy­droxy H atoms. Grey mol­ecules correspond to the main part *A*/*B* (occupancy 0.74) and gold mol­ecules to the minor part *C*/*D* (occupancy 0.26); hy­droxy O atoms are represented with magenta ellipsoids. Two tetra­mers are displayed in a projection along the threefold crystallographic axis. The difference map is plotted at the 0.33 e Å^−3^ level (green wire for Δρ > 0 and red wire for Δρ < 0; Dolomanov *et al.*, 2009 ▸). Note how most of the positive residuals are concentrated within the cavity delimited by the eight clustered O atoms. The inset is the crystal used for data collection. Note the triangular face on the top of the crystal, corresponding to the (003) face. (Right) Final model, including 24 disordered hy­droxy H atoms, shown as green spheres with a radius corresponding to 33% of the van der Waals radius. All C and O atoms are displayed with displacement ellipsoids at the 20% probability level (*Mercury*; Macrae *et al.*, 2008 ▸).

Crystal data, data collection and structure refinement details are summarized in Table 1 ▸ for the two different crystals obtained from a single crystallization batch, but diffracted at different temperatures, *i.e.* 153 and 295 K. The disorder in the asymmetric unit was solved using the low-temperature data set, and all phenyl rings were restrained to be flat, with standard deviations of 0.1 Å^3^. Additionally, the phenyl rings in the minor part (mol­ecules *C* and *D*) were restrained to have 1,3 distances similar to those in the corresponding rings of the disordered counterpart (mol­ecules *A* and *B*), within standard deviations of 0.02 Å. H atoms in the phenyl rings were placed in idealized positions and refined as riding to their carrier C atoms, with *U*_iso_(H) = 1.2*U*_eq_(C). Hy­droxy H atoms were found in a difference map (Fig. 2 ▸, left) and refined with *U*_iso_(H) = 1.5*U*_eq_(O). Atoms H1*A* and H1*C* are disordered by symmetry, and their site occupancies were fixed as one-third of the occupancy of the part to which they belong. The hy­droxy H atoms for mol­ecules in general positions are disordered over sites H1*BA*, H1*BB* and H1*BC* for mol­ecule *B*, and H1*DA*, H1*DB* and H1*DC* for mol­ecule *D*, and the occupancy for each site was also fixed as one-third of the occupancy of the part to which it belongs. The geometry of the C—O—H groups was first restrained to a sensible target, by restraining distances to O—H = 0.85 (1) Å and C⋯H = 1.93 (2) Å in mol­ecules *A* and *C*; for mol­ecules *B* and *D*, the applied restraints were O—H = *d*, H⋯H = (8/3)^1/2^ × *d* and C⋯H = 2.27 × *d*, where *d* is a common free variable. Standard deviations for these restraints were 0.02, 0.03 and 0.03 Å, respectively. After convergence, the positions of all hy­droxy H atoms were fixed, and these atoms were refined as riding on their carrier O atoms. The final model for the complete structure at 153 K was refined against data collected at 295 K, with an extra restraint: in the minor-disordered part (mol­ecules *C* and *D*), rigid-bond restraints were applied with standard deviations of 0.004 Å for the 1,2 and 1,3 distances (Thorn *et al.*, 2012 ▸; Sheldrick, 2015*b* ▸).

## Results and discussion   

The asymmetric unit of the trigonal cell includes two disordered parts with site-occupancy factors converging at 153 K towards 0.7436 (17) (mol­ecules *A* and *B* hereafter) and 0.2564 (17) (mol­ecules *C* and *D* hereafter), close to the occupancies reported by Ferguson *et al.* of 0.71 and 0.29. Each part contains two independent mol­ecules, one of which has the σ C—O bond lying on the threefold axis in the space group *R* 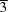, while the other is located in a general position. The arrangement of these four disordered mol­ecules generates overlapped phenyl rings in the asymmetric unit, making the refinement of displacement parameters a tedious task (see §2.2). However, the mol­ecular structure based on data collected at 153 K can be considered as satisfactory, although the refinement was carried out with restrained geometry for the phenyl rings. The refinement based on data collected at 295 K is not as easy, since the scattering power of the crystal decreases dramatically: the fraction of observed data [*I*/σ(*I*) > 2] drops from 50% at 153 K to 36% at 295 K. However, the structure is essentially unmodified, and occupancies for the disordered parts refined to 0.761 (3) and 0.239 (3). At both temperatures, all non-H atoms could be refined anisotropically (see Fig. 2 ▸, right), after which phenyl H atoms were placed in idealized positions.

**Figure 3:**
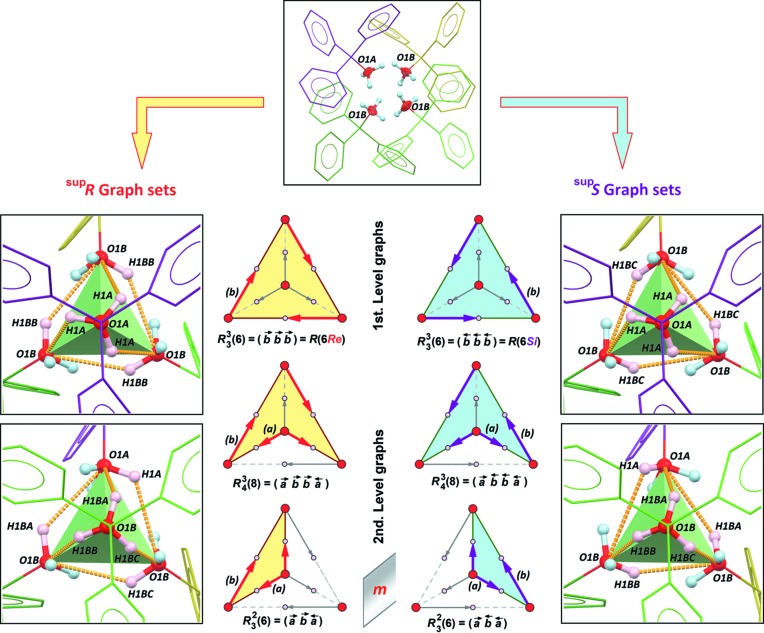
Hydrogen-bonding schemes in the tetra­mer formed by *A*/*B* mol­ecules. The top figure shows the arrangement of the four mol­ecules and the 12 hy­droxy H-atom sites. The left and right panels represent right- (^sup^ *R*) and left-handed (^sup^ *S*) supra­molecular clusters, respectively. The first figure is oriented down the crystallographic threefold axis and the other is oriented down a noncrystallographic threefold axis. Each cluster comprises six hydrogen bonds (dashed gold bonds), involving six H-atom sites (pink H atoms). Topological graphs *G*(4,6) for supra­molecular clusters based on O—H⋯O hydrogen bonds are represented in the centre. Nodes are represented as red balls (O atoms). Arrows forming a ring *R_d_^a^*(*n*) are stacked over O—H covalent bonds and oriented in the direction *d*→*a*, where *d* is the donor and *a* the acceptor for a hydrogen bond. Arrows involved in a ring are shown in bold, while those not participating in a ring are greyed out. Polygons delimited by *R* rings in the 2-space are coloured yellow and blue for ^sup^ *R* and ^sup^ *S* clusters, respectively, and rings in the projection plane are read clockwise in all cases. For the first-level graphs, *Re* stands for *Rectus* and *Si* for *Sinister*. Note that all figures on the left are mirror images of the figures on the right, including descriptors of the *R* rings.

The localization of the hy­droxy H atoms was far more complex. A difference map using room-temperature data is useless, in contrast to the map computed with data collected at low temperature. At 153 K, most of the positive residuals are found close to the O atoms (Fig. 2 ▸, left), allowing the determination of sensible coordinates for H atoms disordered by symmetry (H1*A* and H1*C* for mol­ecules *A* and *C* in special positions). At this point, the highest residuals are found close to O1*B*, forming a tetra­hedral geometry with O1*B*; although mol­ecule *B* is located in a general position, the O—H group emulates the disorder imposed by symmetry in mol­ecule *A* (see top inset in Fig. 3 ▸). The same situation is repeated for mol­ecule *D*, with much smaller residuals because this mol­ecule belongs to the minor part of the disordered asymmetric unit. Ultimately, all the mol­ecules in the crystal have their hy­droxy H atoms disordered over three sites, and once the asymmetric unit is expanded to tetra­mers, eight independent mol­ecules are clustered in such a way that the cavity delimited by eight O atoms is filled with 24 sites for disordered hy­droxy H atoms (Fig. 2 ▸, right). Each C—O—H group can also be seen as a rigid group rotating about its C—O axis, producing for the H atom an electron density smeared out over a ring; nevertheless, the free rotation should be hindered through the formation of weak hydrogen bonds (Schröder *et al.*, 2004 ▸). Such a description would be consistent with ^2^H NMR spectroscopy experiments carried out on Ph_3_COD, showing that each hy­droxy group is dynamic by rotation about the C—OD bond, on the 10^−3^ to 10^−8^ s time scale (Aliev *et al.*, 1998 ▸).

All OH groups behave as donor groups for inter­molecular O—H⋯O hydrogen bonds. For the main part *A*/*B*, with occupancy = 0.74, three *B* mol­ecules placed close to the threefold axis are connected to form an 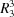
(6) ring, corresponding to a first-level graph with H1*BB* as donor (Table 2 ▸, entry 3). This motif is repeated with H1*BC* (Table 2 ▸, entry 4), however, if the crystal orientation is preserved, this ring motif is enanti­omorphic with the previous one. Finally, the third disordered site for the hy­droxy H atom, H1*BA*, is engaged in a second-level graph with O1*A* as acceptor (Table 2 ▸, entry 2), giving a ring motif 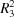
(6). Site O1*A* also serves as a donor, forming three symmetry-equivalent O1*A*—H1*A*⋯O1*B* hydro­gen bonds (Table 2 ▸, entry 1), and is involved in the largest rings, 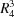
(8). All rings are depicted in Fig. 3 ▸, along with schematic representations of the corresponding graphs and their pathways [*i.e.* the *constructor graphs* and the *qualitative descriptors*, in the terminology coined by Motherwell *et al.* (1999 ▸)].

**Figure 4:**
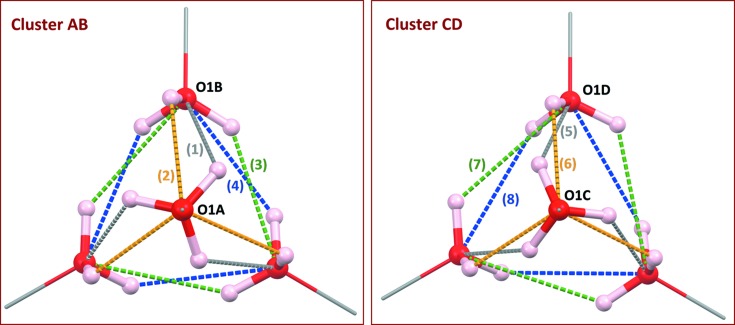
Complete set of hydrogen bonds, represented as dashed lines, in the tetra­mer formed by mol­ecules *A* and *B* (left), and in the tetra­mer formed by mol­ecules *C* and *D* (right). Figures are oriented down the crystallographic threefold axis. Labels (1)⋯(8) on hydrogen bonds indicate the entry in Table 2 ▸. Each cluster has four independent bonds, affording 12 bonds for the tetra­mer, consistent with the *C*_3_ point symmetry of the tetra­mer. These figures can be compared to the model published in 1999 (see Fig. 4 ▸ in Serrano-González *et al.*, 1999 ▸).

The tetra­mer based on *A* and *B* mol­ecules includes 12 hydrogen bonds. Each disordered hy­droxy H atom is engaged in a single hydrogen bond, and each O atom serves three times as acceptor (Fig. 4 ▸, left). The hydrogen-bonded supra­molecular cluster formed in the tetra­mer is associated with a topological graph embedded in 3-space, *i.e.* *G*(4,6) = [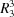
(6)
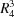
(8)
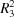
(6)], for which the faces are the *R*(*n*) rings described in Fig. 3 ▸. In the parlance of graph theory, the finite directed graph *G*(4,6) based on the ‘*pyramidal tetra­mer*’ mentioned by Ferguson *et al.* is regular, complete and intrinsically chiral (Flapan, 1995 ▸). The four nodes for *G*(4,6) are provided by four mol­ecules (or four hy­droxy O atoms for simplicity) and the six arrows are oriented O—H⋯O hydrogen bonds, the tail of the arrow being the donor OH group and the head being the acceptor O atom. It is noteworthy that for each right-handed *R*(*n*) ring, there is one related left-handed ring, as illustrated in Fig. 3 ▸. For example, in the first-level 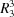
(6) subgraph embedded in the 2-space, arrows rotate clockwise around the crystallographic threefold axis for the ring including arrows O1*B*—H1*BB*⋯O1*B* (*Rectus* face for topological graph *G*), and anti­clockwise for the ring including arrows O1*B*—H1*BC*⋯O1*B* (*Sinister* face for topological graph *G*).

A topologically isomorphous graph *G*′(4,6) can be built with the minor part of the asymmetric unit, including hydrogen bonds similar to those described for the main tetra­mer, although the relative positions of the 12 H-atom sites is modified through a small rotation around the C1*C*—O1*C* axis (Fig. 4 ▸, right). Therefore, the mixture of eight mol­ecules built on the asymmetric unit, as represented in Fig. 2 ▸, affords a racemic mixture of supra­molecular enanti­omorphic tetra­mers ^sup^ *R* and ^sup^ *S* built on 24 hydrogen bonds. Obviously, the mol­ecules themselves are achiral, but the supra­molecular chirality results from the asymmetric configuration of the hydrogen bonds (Sasaki *et al.*, 2014 ▸). Neither the unit cell nor the crystal are chiral objects, since the mol­ecule crystallizes in a centrosymmetric space group, *R* 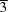.

**Figure 5:**
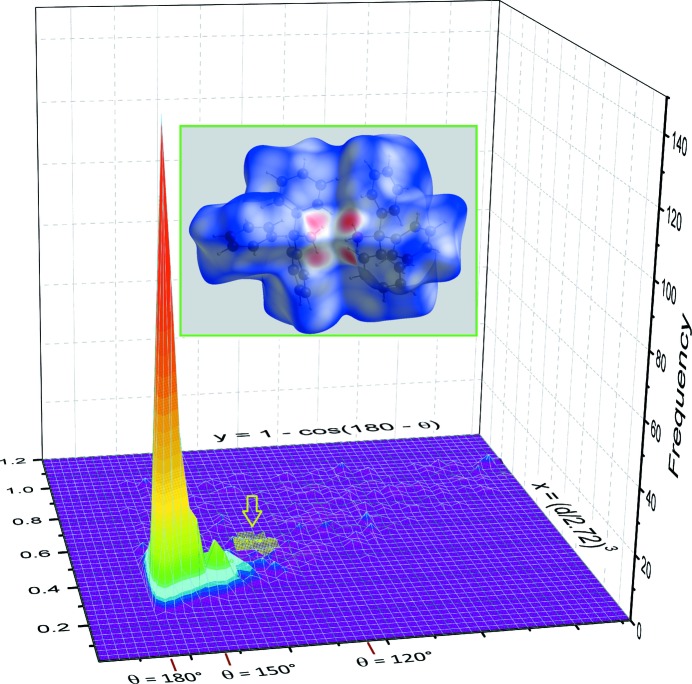
Histogram of the O—H⋯O inter­molecular hydrogen-bond geometry in the crystal structures of tertiary alcohols, in spherical polar coordinates (*x*, *y*), with the CSD frequency shown in the third dimension (OriginLab, 2012 ▸). A log_10_ rainbow colour scheme is used to highlight small frequencies. Some values for the O—H⋯O angles θ are reported on axis *y*, for reference. Note the similarity of the distribution with that depicted in the CCDC article about the directionality of hydrogen bonds (Wood *et al.*, 2009 ▸; see Fig. 2 ▸ in this article). The green patch marked with an arrow in the (*x*, *y*) plane defines the area for hydrogen bonds in the *A*/*B* tetra­meric cluster of the title compound at 153 K. The inset shows the Hirshfeld surface mapped over *d* (−1 to 1 Å) for the *A*/*B* mol­ecules at 153 K (Turner *et al.*, 2017 ▸); each of the four red patches on the surface is related to a single node for the topological graph *G*(4,6) of the *A*/*B* tetra­mer (see Fig. 3 ▸).

The set of 24 hydrogen bonds depicted in Fig. 4 ▸ comprises only weak hydrogen bonds, with H⋯O separations in the range 2.21–2.38 Å and O—H⋯O angles far from linearity, in the range 121.4–138.2°, at 153 K. The refinement using room-temperature data indicates that the supra­molecular chiral clusters are retained, although hydrogen bonds are slightly weakened by *ca* 0.06 Å for H⋯O separations (compare Tables 2 ▸ and 3 ▸). These geometric parameters were compared with those found in other supra­molecular networks formed in the crystalline state by tertiary alcohols, using the methodology developed at the CCDC (Wood *et al.*, 2009 ▸). Parameters for inter­molecular O—H⋯O contacts in tertiary alcohols were retrieved from the current release of the CSD, omitting disordered, polymeric and ionic structures. Hy­droxy H-atom positions were normalized within *ConQuest* to O—H = 0.993 Å, in order to avoid systematic errors in contact distances (Bruno *et al.*, 2002 ▸). The search was limited to organic compounds not flagged with errors, and reported with *R*_1_ < 0.075, affording 1215 hits, corresponding to 1812 raw data (*d*, θ), where *d* is the H⋯O distance and θ is the O—H⋯O angle. Only contacts with *d* shorter than the van der Waals (vdW) distance were retained [*r*_vdW_(H) + *r*_vdW_(O) = 2.72 Å; Bondi, 1964 ▸]. Data were converted into spherical polar coordinates (*x*, *y*), where *x* = (*d*/2.72)^3^ and *y* = 1 − cos (180 − θ), assuming that *d* and θ are expressed in Å and °, respectively (Lommerse *et al.*, 1996 ▸). A two-dimensional (2D) frequency binning histogram representation of the (*x*, *y*) distribution shows a sharp peak about (*d*, θ) = (1.82 Å, 180°), as expected for O—H⋯O hydrogen bonds (Fig. 5 ▸). A significant frequency is still observed about (*d*, θ) = (1.87 Å, 145°). Outside these well-defined territories, the observed frequency collapses.

Inter­estingly, the title compound displays O—H⋯O contacts on the borderline between truly hydrogen-bonded alcohols and the near-zero frequency area (see green patch on the ground level in Fig. 5 ▸). However, although of very limited strength, hydrogen bonds in the tetra­mers depicted in Fig. 3 ▸ are genuinely present, as reflected in the Hirshfeld surface for any pair of mol­ecules involved in a tetra­meric supra­molecular cluster (Fig. 5 ▸, inset). In other words, tri­phenyl­methanol could represent the boundary between hydrogen-bonded and non-hydrogen-bonded tertiary alcohols. This also opens the possibility that a phase transition occurs for tri­phenyl­methanol somewhere between *T* = 293 and 433 K (melting point), if thermal energy *kT* is able to dismantle the network of weak hydrogen bonds.

In order to evaluate the stability of the noncovalent bonds in this crystal, George Ferguson and co-workers came across a more chemical strategy, by determining the crystal structures of mol­ecules isoelectronic with tri­phenyl­methanol (Glidewell & Ferguson, 1994 ▸). Their hypothesis was that ‘with only modest changes in the steric demands at the unique central C atom, while keeping the number of hydrogen-bond donors and acceptors unchanged, the patterns of hydrogen bonding can be altered drastically’. Indeed, diphen­yl(pyridin-4-yl)methanol has a simple achiral supra­molecular structure based on *C*(7) chains. For tri­phenyl­methanamine, two polymorphic forms have been described: the ortho­rhom­bic phase does not form hydrogen bonds at all (Glidewell & Ferguson, 1994 ▸), while the triclinic phase features dimers through the formation of N—H⋯N hydrogen bonds, due to the statistical disordering of the amino H atoms (Khrustalev *et al.*, 2009 ▸; Schulz *et al.*, 2013 ▸). The NH_2_ group then displays a geometry reminiscent of that of the OH groups in tri­phenyl­methanol. However, no chiral supra­molecular clusters are formed with the amine, and the asymmetric unit includes a single nondisordered mol­ecule. A rhombohedral polymorph for this amine has also been deposited recently; unfortunately, after inspection of this structure, it turns out that the formula is wrong: the diffracted crystal was almost certainly tri­phenyl­methanol (Bagchi *et al.*, 2014 ▸; *R* 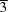 space group, *T* = 298 K, *R*_1_ = 10%). In the opposite direction, strong O—H⋯O hydrogen bonds can be restored in sterically hindered tertiary alcohols by just adding a methyl­ene group: in the crystal structure of tri­phenyl­ethanol, Ph_3_CCH_2_OH (nondisordered *P*2_1_/*c* crystal, *Z*′ = 2; Ferguson *et al.*, 1994 ▸), mol­ecules aggregate into discrete achiral 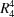
(8) rings. In comparison with tri­phenyl­methanol, the prochiral character of the supra­molecular structure is lost, and the compound lies within a sharp peak of ‘normal’ crystal structures in a (*x*, *y*) distribution similar to that depicted for tertiary alcohols in Fig. 5 ▸.

## Concluding remarks   

Tri­phenyl­methanol is a small simple mol­ecule with a structure unexpectedly difficult to refine compared, for example, to that of tri­phenyl­silanol (Bowes *et al.*, 2002 ▸). It is worthwhile to consider the evolution of X-ray diffractometry over the last 25 years, using tri­phenyl­methanol as a benchmark (Table 4 ▸; data at room temperature were retained in order to avoid biases). The time taken for data collection is almost identical in the three cases, *ca* 20–30 h, and there is little doubt that the diffracted samples were of similar quality. Over time, a steady progress is noted. Development of new technologies for the detection of scattered X-ray photons seems to be the key point, in such a way that location of tiny fractions of electrons in the crystal space is now routinely affordable, outside the multipole density formalism. There is indeed a consensus that the hybrid pixel detectors with CdTe or GaAs sensors represent the ultimate state-of-art technology in this field, since they detect all scattered X-rays, with 100% efficiency and without any noise (Allé *et al.*, 2016 ▸). For the herein presented study, a Pilatus detector was used, with a 1000 µm-thick silicon sensor, which has a quantum efficiency of 50% for 22.2 keV photons (Ag *K*α radiation).

## Supplementary Material

Crystal structure: contains datablock(s) 153K_data, 295K_data, global. DOI: 10.1107/S2053229619010714/cu3146sup1.cif

Structure factors: contains datablock(s) 153K_data. DOI: 10.1107/S2053229619010714/cu3146153K_datasup2.hkl

Structure factors: contains datablock(s) 295K_data. DOI: 10.1107/S2053229619010714/cu3146295K_datasup3.hkl

Supporting information file. DOI: 10.1107/S2053229619010714/cu3146153K_datasup4.cml

CCDC references: 1944593, 1944592

## Figures and Tables

**Table 1:** Experimental details For both determinations: C_19_H_16_O, *M*_r_ = 260.32, trigonal, *R* 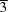:*H*, *Z* = 24. Experiments were carried out with Ag *K*α radiation (λ = 0.56083 Å) using a Stoe Stadivari diffractometer. Absorption was corrected for by multi-scan methods (*X-AREA*; Stoe & Cie, 2018 ▸). Refinement was on 483 parameters. H-atom parameters were constrained.

	153 K data	295 K data
Crystal data		
Temperature (K)	153	295
*a*, *c* (Å)	19.1399 (5), 26.7399 (9)	19.3309 (8), 26.8542 (11)
*V* (Å^3^)	8483.4 (5)	8690.5 (8)
μ (mm^−1^)	0.05	0.05
Crystal size (mm)	0.33 × 0.29 × 0.25	0.38 × 0.33 × 0.33

Data collection		
*T*_min_, *T*_max_	0.419, 1.000	0.558, 1.000
No. of measured, independent and observed [*I* > 2σ(*I*)] reflections	99452, 4399, 2222	72080, 4523, 1656
*R*_int_	0.069	0.108
(sin θ/λ)_max_ (Å^−1^)	0.653	0.653

Refinement		
*R*[*F* ^2^ > 2σ(*F* ^2^)], *wR*(*F* ^2^), *S*	0.052, 0.167, 1.00	0.063, 0.242, 0.99
No. of reflections	4399	4523
No. of restraints	72	279
Δρ_max_, Δρ_min_ (e Å^−3^)	0.18, −0.14	0.11, −0.14

Computer programs: *X-AREA* (Stoe & Cie, 2018 ▸), *SHELXT2018* (Sheldrick, 2015*a* ▸), *SHELXL2018* (Sheldrick, 2015*b* ▸), *XP* in *SHELXTL-Plus* (Sheldrick, 2008 ▸), *Mercury* (Macrae *et al.*, 2008 ▸), *OLEX2* (Dolomanov *et al.*, 2009 ▸) and *publCIF* (Westrip, 2010 ▸).

**Table 2:** Hydrogen-bond geometry (Å, °) for the 153 K[Chem scheme1] data

*D*—H⋯*A*	*D*—H	H⋯*A*	*D*⋯*A*	*D*—H⋯*A*
O1*A*—H1*A*⋯O1*B*	0.85	2.33	2.869 (3)	121
O1*B*—H1*BA*⋯O1*A*	0.84	2.27	2.869 (3)	129
O1*B*—H1*BB*⋯O1*B* ^i^	0.82	2.21	2.869 (3)	138
O1*B*—H1*BC*⋯O1*B* ^ii^	0.86	2.24	2.869 (3)	130
O1*C*—H1*C*⋯O1*D*	0.85	2.22	2.856 (7)	131
O1*D*—H1*DA*⋯O1*C*	0.84	2.24	2.856 (7)	131
O1*D*—H1*DB*⋯O1*D* ^ii^	0.82	2.38	2.951 (8)	127
O1*D*—H1*DC*⋯O1*D* ^i^	0.85	2.33	2.951 (8)	131

Symmetry codes: (i) 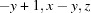; (ii) 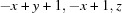.

**Table 3:** Hydrogen-bond geometry (Å, °) for the 295 K data[Chem scheme1]

*D*—H⋯*A*	*D*—H	H⋯*A*	*D*⋯*A*	*D*—H⋯*A*
O1*A*—H1*A*⋯O1*B*	0.85	2.56	2.917 (3)	106
O1*B*—H1*BA*⋯O1*A*	0.82	2.27	2.917 (3)	136
O1*B*—H1*BB*⋯O1*B* ^i^	0.82	2.31	2.912 (3)	131
O1*B*—H1*BC*⋯O1*B* ^ii^	0.85	2.31	2.912 (4)	128
O1*C*—H1*C*⋯O1*D*	0.85	2.27	2.916 (13)	133
O1*D*—H1*DA*⋯O1*C*	0.83	2.26	2.916 (13)	136
O1*D*—H1*DB*⋯O1*D* ^ii^	0.83	2.35	2.947 (13)	130
O1*D*—H1*DC*⋯O1*D* ^i^	0.84	2.33	2.947 (13)	132

Symmetry codes: (i) 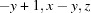; (ii) 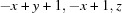.

**Table 4:** Comparison between the three X-ray structures of tri­phenyl­methanol determined at room temperature

Date of publication	1992^*a*^	1999^*b*^	2019^*c*^
Diffractometer	CAD-4	R-Axis II	Stadivari
Detector	NaI scintillator	Image plate	HPAD^*d*^
*T* (K)	294	293	295
No. independent reflections	2467	3448	4523
Refined parameters	253	322	483
Data resolution (Å)	0.89	0.82	0.77
Range for σ(C—C)	0.040–0.007 Å	–	0.020–0.003 Å

Notes and references: (*a*) Ferguson *et al.* (1992 ▸); (*b*) Serrano-González *et al.* (1999 ▸); (*c*) this work; (*d*) Hybrid Pixel Array Detector.
